# Supplementary material for: Modeling glioblastoma heterogeneity as a dynamic network of cell states
Source: Mol Syst Biol. 2021 Sep 16;17(9):e10105. doi: 10.15252/msb.202010105 (PMC8444284; doi:10.15252/msb.202010105)
Supplement: Supplementary file 6 — Source Data for Figure 5 [file MSB-17-e10105-s004.zip › Figure5A_sourcedata/GSEA_3071/hallmarks_stateIII.GseaPreranked.1623742540963/HALLMARK_OXIDATIVE_PHOSPHORYLATION.html]

Details for gene set HALLMARK\_OXIDATIVE\_PHOSPHORYLATION[GSEA]

|  || Dataset | state33071 |
| Phenotype | NoPhenotypeAvailable |
| Upregulated in class | na\_pos |
| GeneSet | HALLMARK\_OXIDATIVE\_PHOSPHORYLATION |
| Enrichment Score (ES) | 0.7206378 |
| Normalized Enrichment Score (NES) | 6.6186976 |
| Nominal p-value | 0.0 |
| FDR q-value | 0.0 |
| FWER p-Value | 0.0 |
Table: GSEA Results Summary

  

Fig 1: Enrichment plot: HALLMARK\_OXIDATIVE\_PHOSPHORYLATION      
 Profile of the Running ES Score & Positions of GeneSet Members on the Rank Ordered List

  

| PROBE | GENE SYMBOL | GENE\_TITLE | RANK IN GENE LIST | RANK METRIC SCORE | RUNNING ES | CORE ENRICHMENT || 1 | UQCRQ |  |  | 13 | 0.574 | 0.0261 | Yes |
| 2 | ATP5PD |  |  | 37 | 0.486 | 0.0451 | Yes |
| 3 | NDUFB2 |  |  | 52 | 0.460 | 0.0651 | Yes |
| 4 | NDUFA6 |  |  | 54 | 0.458 | 0.0883 | Yes |
| 5 | CYCS |  |  | 59 | 0.453 | 0.1105 | Yes |
| 6 | COX8A |  |  | 63 | 0.448 | 0.1328 | Yes |
| 7 | UQCR10 |  |  | 89 | 0.421 | 0.1479 | Yes |
| 8 | ATP5ME |  |  | 91 | 0.416 | 0.1690 | Yes |
| 9 | VDAC1 |  |  | 98 | 0.413 | 0.1887 | Yes |
| 10 | ATP5MF |  |  | 100 | 0.411 | 0.2095 | Yes |
| 11 | UQCRB |  |  | 117 | 0.398 | 0.2258 | Yes |
| 12 | COX5A |  |  | 131 | 0.389 | 0.2425 | Yes |
| 13 | COX6B1 |  |  | 134 | 0.387 | 0.2618 | Yes |
| 14 | COX7B |  |  | 135 | 0.386 | 0.2816 | Yes |
| 15 | NDUFA4 |  |  | 140 | 0.382 | 0.3002 | Yes |
| 16 | NDUFA1 |  |  | 143 | 0.381 | 0.3192 | Yes |
| 17 | COX6A1 |  |  | 145 | 0.379 | 0.3384 | Yes |
| 18 | ATP5MG |  |  | 146 | 0.377 | 0.3578 | Yes |
| 19 | COX7C |  |  | 170 | 0.362 | 0.3704 | Yes |
| 20 | ATP5PF |  |  | 191 | 0.346 | 0.3830 | Yes |
| 21 | NDUFB6 |  |  | 199 | 0.339 | 0.3986 | Yes |
| 22 | ATP5F1E |  |  | 207 | 0.334 | 0.4139 | Yes |
| 23 | ATP5MC1 |  |  | 208 | 0.334 | 0.4311 | Yes |
| 24 | COX4I1 |  |  | 223 | 0.326 | 0.4442 | Yes |
| 25 | NDUFB1 |  |  | 224 | 0.326 | 0.4609 | Yes |
| 26 | UQCR11 |  |  | 225 | 0.326 | 0.4776 | Yes |
| 27 | ATP6V1F |  |  | 229 | 0.323 | 0.4935 | Yes |
| 28 | NDUFA2 |  |  | 239 | 0.315 | 0.5073 | Yes |
| 29 | NDUFA3 |  |  | 260 | 0.305 | 0.5178 | Yes |
| 30 | COX5B |  |  | 280 | 0.297 | 0.5281 | Yes |
| 31 | ATP5MC2 |  |  | 282 | 0.296 | 0.5431 | Yes |
| 32 | NDUFC1 |  |  | 284 | 0.296 | 0.5580 | Yes |
| 33 | TOMM22 |  |  | 290 | 0.294 | 0.5718 | Yes |
| 34 | TIMM10 |  |  | 304 | 0.290 | 0.5833 | Yes |
| 35 | NDUFB3 |  |  | 312 | 0.288 | 0.5963 | Yes |
| 36 | TIMM8B |  |  | 322 | 0.286 | 0.6087 | Yes |
| 37 | SDHB |  |  | 323 | 0.286 | 0.6233 | Yes |
| 38 | ATP5MC3 |  |  | 334 | 0.283 | 0.6352 | Yes |
| 39 | ATP5PO |  |  | 337 | 0.282 | 0.6492 | Yes |
| 40 | NDUFA5 |  |  | 346 | 0.278 | 0.6614 | Yes |
| 41 | COX17 |  |  | 368 | 0.270 | 0.6698 | Yes |
| 42 | MDH2 |  |  | 393 | 0.262 | 0.6771 | Yes |
| 43 | HADHA |  |  | 408 | 0.257 | 0.6866 | Yes |
| 44 | NDUFB4 |  |  | 427 | 0.252 | 0.6949 | Yes |
| 45 | NDUFB8 |  |  | 428 | 0.251 | 0.7078 | Yes |
| 46 | UQCRH |  |  | 429 | 0.250 | 0.7206 | Yes |
| 47 | ETFDH |  |  | 798 | -0.269 | 0.6395 | No |
| 48 | MTRF1 |  |  | 949 | -0.277 | 0.6149 | No |
| 49 | MTRR |  |  | 1166 | -0.288 | 0.5740 | No |
| 50 | ATP6AP1 |  |  | 1471 | -0.304 | 0.5111 | No |
| 51 | SLC25A12 |  |  | 1712 | -0.319 | 0.4656 | No |
| 52 | CPT1A |  |  | 1816 | -0.325 | 0.4557 | No |
| 53 | MAOB |  |  | 2151 | -0.345 | 0.3872 | No |
| 54 | ATP1B1 |  |  | 2865 | -0.405 | 0.2239 | No |
| 55 | RETSAT |  |  | 3245 | -0.450 | 0.1492 | No |
| 56 | PDP1 |  |  | 3555 | -0.528 | 0.0965 | No |
Table: GSEA details [plain text format]

  

Fig 2: HALLMARK\_OXIDATIVE\_PHOSPHORYLATION: Random ES distribution      
 Gene set null distribution of ES for **HALLMARK\_OXIDATIVE\_PHOSPHORYLATION**
